# DNA-Mediated Patterning of Single Quantum Dot Nanoarrays: A Reusable Platform for Single-Molecule Control

**DOI:** 10.1038/srep45591

**Published:** 2017-03-28

**Authors:** Da Huang, Mark Freeley, Matteo Palma

**Affiliations:** 1 School of Biological and Chemical Sciences, Materials Research Institute, and Institute of Bioengineering, Queen Mary University of London, Mile End Road, London E1 4NS, UK

## Abstract

We present a facile strategy of general applicability for the assembly of individual nanoscale moieties in array configurations with single-molecule control. Combining the programming ability of DNA as a scaffolding material with a one-step lithographic process, we demonstrate the patterning of single quantum dots (QDs) at predefined locations on silicon and transparent glass surfaces: as proof of concept, clusters of either one, two, or three QDs were assembled in highly uniform arrays with a 60 nm interdot spacing within each cluster. Notably, the platform developed is reusable after a simple cleaning process and can be designed to exhibit different geometrical arrangements.

The tunable emission, efficient broadband light harvesting capability, and solution processability of semiconductor quantum dots (QDs) make them ideal building blocks for new generation nanoelectronic and nanoplasmonic devices^1^,^2^. QD-based solid-state platforms are indeed of importance in photovoltaics^3^,^4^,^5^,^6^,^7^,^8^ and quantum information technology^9^,^10^,^11^,^12^, as light emitting diodes and photodetectors^13^,^14^,^15^,^16^, as well as for quantum optics experiments^17^,^18^,^19^,^20^,^21^.

A key requirement for all the aforementioned applications, and for the future miniaturization of photonic integrated devices, is the controlled organization of QDs from solution to surfaces. In this regard, the precise patterning of colloidal semiconductor nanocrystals into hierarchical structures has attracted substantial research interest in recent years. Various strategies have been presented for the geometrically controlled assembly of QDs on different substrates. Lithographic nanopatterning has provided a valuable approach for the formation of assemblies/clusters down to 15 nm in size^22^,^23^,^24^,^25^,^26^. Additionally, biologically inspired scaffolds have shown promise for the formation of arrays of QDs; examples include: DNA-mediated self-assembly^27^,^28^,^29^,^30^,^31^, genetically engineered bacteriophage viruses^32^, host-polymers^33^,^34^, and the use of solid-binding peptide linkers^35^.

Notably, significant effort has been devoted to achieving individual QD control, that in turn is of particular interest for Quantum Electrodynamics (QED) investigations^17^,^18^,^19^,^20^, e.g. the coupling of QDs to nanocavities, for the advance of nanoscale quantum emitters^21^,^36^, and more generally for the development of single-QD based optoelectronic devices. The highest level of control attained to date in the organization of individual solution-processable QDs was demonstrated via the formation of predominantly single-QD nanoarrays, but only when the nanocrystals were coupled to pre-patterned metal nanodots^37^. Differently, Xie *et al*. have very recently obtained the positioning of QDs directly on silicon, but with a yield of single-dot patterning of only 40%^38^, and without the ability to control the assembly of multiple individual QDs per array’s location.

Herein we present a facile strategy to control the number (e.g. one, two, or three) and position of single QDs at predefined locations in nanoarrays, with nanoscale interdot spacing. The approach presented is of general applicability for the assembly of nanostructures in highly uniform nanoarrays with single-molecule control. Our strategy combines the programming ability of DNA as a scaffolding material^39^,^40^,^41^,^42^,^43^, with a one-step lithographic process. As a proof of concept, we achieved the immobilization of individual nanocrystals in nanoarrays on both silicon and transparent glass surfaces, with a 60 nm interdot spacing in clusters of two and three QDs, and with up to 82% yield in single-QD patterning. Additionally, the platform developed is reusable after a simple cleaning process and can be designed to exhibit different geometrical arrangements.

## Results

For our studies we employed triangular DNA origami structures of 120 nm by side^44^ as molecular breadboards for the assembly of individual CdSe/ZnS core/shell QDs [see the Supplementary Information (SI) and Figure SI1]. Binding sites for (streptavidin-coated) nanocrystals were incorporated along the axis of the DNA nanostructure using biotin-labelled staple strands (see the SI and Figure SI2)^29^,^30^,^42^,^45^. This allowed us to design individual DNA origami scaffolds for the tethering of either one, two, or three QDs per DNA nanostructure, with a 60 nm interdot spacing.

**Figure 1:**
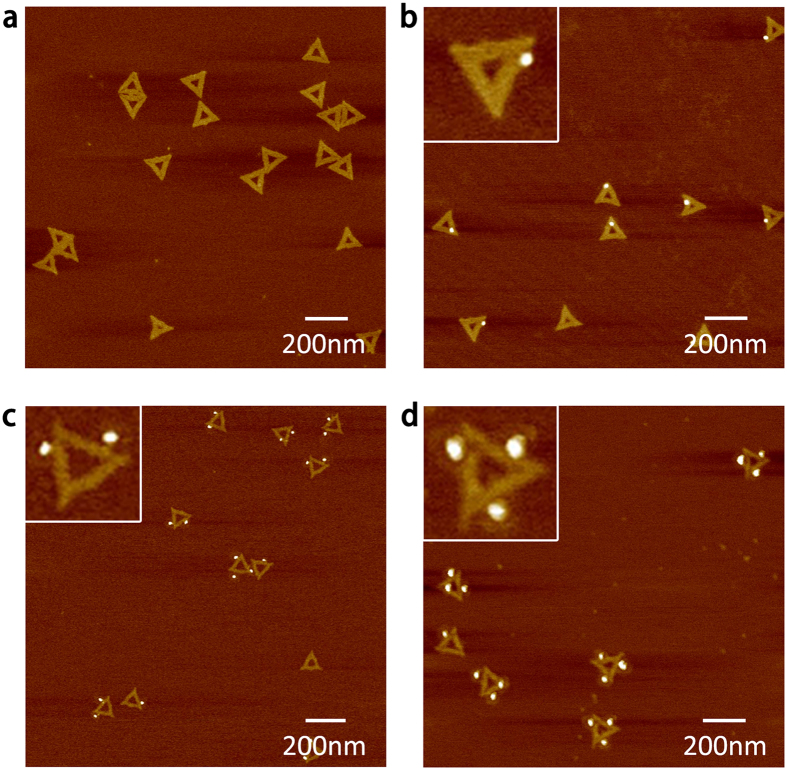
AFM images of triangular DNA origami. (**a**) pristine origami, (**b**) functionalized with one QD, (**c**) functionalized with two QDs, and (**d**) functionalized with three QDs.

Figure 1 shows Atomic Force Microscopy (AFM) images of the triangular DNA origami employed, and the successful organization of individual QDs on the DNA scaffold. The yield of QD attachment on our triangular DNA origami was found to be of 86% for three QDs, 89% for two QDs, and 91% for one QD per origami (see also Figure SI3).

**Figure 2:**
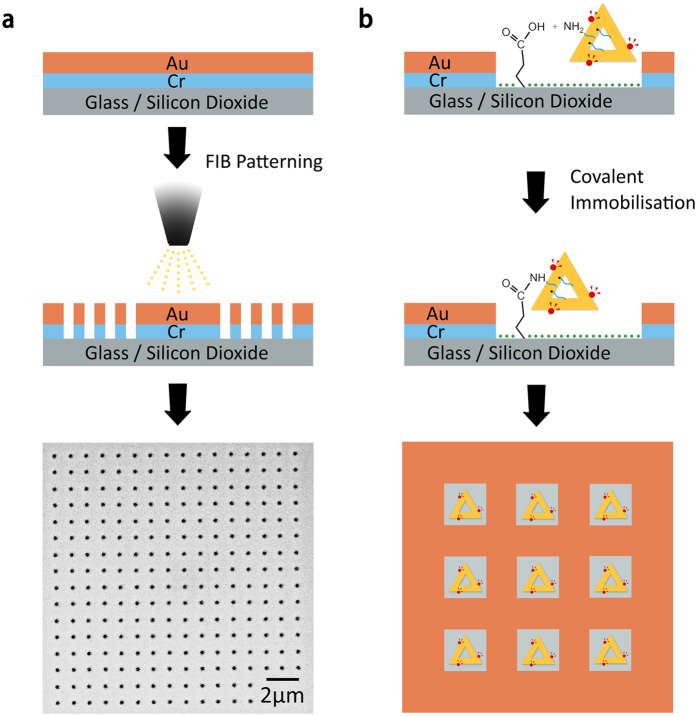
Schematic and images of the patterning and assembly process. (**a**) Schematic of the patterning to fabricate nanoaperture arrays and SEM image of the final substrate; (**b**) Schematic of the covalent immobilisation of amino-terminated and QD-labelled triangular DNA origami on patterned surfaces via amidation reactions.

In order to generate predefined locations for the immobilization of the aforementioned QD-labelled DNA nanostructures from solution to surfaces, we patterned nanoaperture arrays on silicon wafers and transparent insulating glass coverslips. A one-step Focused Ion Beam (FIB) lithography process was employed to selectively fabricate arrays of nanoapertures on metal coated (1.5 nm Cr, 3 nm Au) substrates (see Fig. 2a and the SI). The employed strategy allows for the facile pattering on transparent surfaces and is of general applicability for the concomitant fabrication of cavities in different materials. Moreover, the fabrication can be easily tailored towards inter-aperture spacing of a few μm to prevent any crosstalk between optical signals from neighboring QDs once immobilized on the patterned surface.

The exposed SiO_2_ surface in the fabricated nanoapertures can be chemically modified to covalently tether amino-functionalized moieties, including DNA origami, as previously shown on silicon substrates patterned via electron-beam lithography (EBL)^46^. Briefly, the DNA origami solution was cast on the patterned substrate in the presence of Mg^2+^ (to induce initial physisorption) and carboxyethylsilane. The latter forms carboxylic terminating monolayers on the SiO_2_ surface exposed in the patterned nanoapertures. Standard amide coupling and activating agents (NHS and EDC respectively, see the methods section and the SI for experimental details) were then used to activate the carboxylic groups.

We designed our triangular DNA nanostructures to exhibit 15 amino-terminated DNA strands protruding out of plane of the origami, in addition to the QD-anchoring staple strands (see the SI and Fig. SI-2). Therefore, upon silanisation (with carboxyl groups) of the SiO_2_ surface exposed in the patterned nanoapertures, we covalently tethered our QD-labelled DNA Origami: see Fig. 2b and the SI.

**Figure 3:**
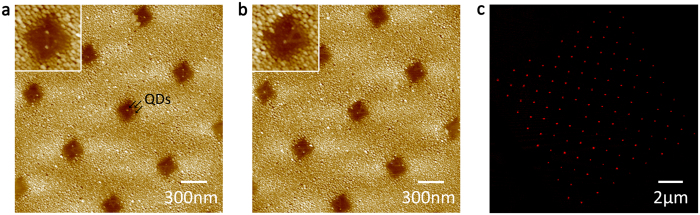
Images of the assembly of QDs in nanoarrays via the covalent immobilisation of QD-labelled triangular DNA origami in pre-patterned nanoapertures. (**a**) AFM image for the case of 3 QDs per origami, and hence per aperture; (**b**) AFM image for the case of 2 QDs; (**c**) Epifluorescence microscopy image of a glass substrate patterned with 3 QDs per 1 μm spaced nanoaperture: each nanoaperture is optically resolvable.

Figure 3 shows AFM images demonstrating the selective placement of three (Fig. 3a) and two (Fig. 3b) QDs per nanoaperture, via the covalent immobilisation of arrays of QD-labelled triangular DNA origami nanostructures on SiO_2_. We have employed nanoapertures of different sizes (easily tailored via FIB patterning), ranging from 120 nm (the size of the DNA triangles) to 250 nm. Notably, even in the larger 250 nm cavities we obtained close to complete immobilisation of a single triangular DNA origami per aperture (90%), rather than multiple (3%), or none (7%). This high yield of one-to-one immobilisation of DNA origami per nanoaperture is most likely due to steric hindrance effects and electrostatic repulsion among the DNA triangles upon their physisorption in the apertures (via a Mg^2+^ bridge) prior to covalent attachment (see also the Methods section and the SI). As a proof of principle, we present here the results obtained with the large apertures because of the higher clarity of the AFM images (see also Figure SI4a and b).

The obtained QD assembly is highly selective as the employed QD-labelled DNA origami do not bind to the metal surface surrounding the apertures. (Non-specific adsorption can be easily minimized by simply rinsing the substrates with buffer solution and DI water after the covalent immobilisation: see the SI). Evidence of this is shown in Fig. 3c, where individual apertures fabricated to exhibit a 1 μm spacing are clearly resolvable via conventional epifluorescence microscopy imaging. This further demonstrates the applicability of the presented strategy to insulating transparent glass coverslips.

**Figure 4:**
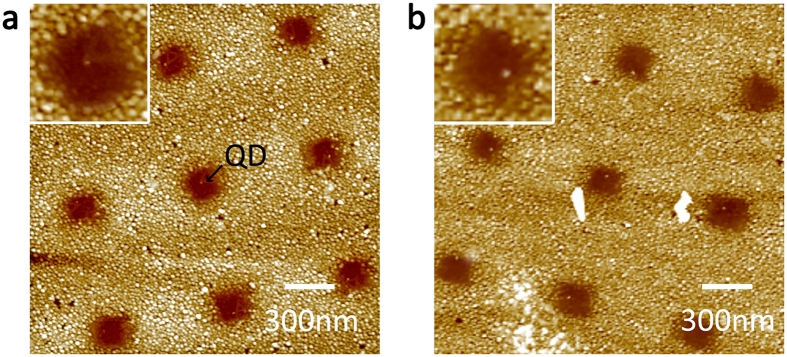
AFM images of single-QD patterning (**a**) single-QD patterning via the covalent immobilisation of triangular DNA origami labelled with one QD: 82% yield; (**b**) AFM image of the same sample after UV/Ozone treatment showing the successful removal of the DNA origami.

Finally, in order to demonstrate single-QD patterning, we fabricated arrays of triangular DNA origami modified with only one QD. Figure 4a shows the successful assembly of single QDs in nanoaperture arrays. The overall yield of single-dot assembly was found to be of 82% over arrays of 64 μm^2^ (four arrays per sample, see also Figure SI4c).

Notably, the platform presented here is reusable by simple Ultraviolet/Ozone treatment of the substrate, followed by a mild ultrasonic cleaning in water, and final rinsing (see the SI). This facile cleaning procedure, allows for the complete removal of DNA nanostructures in the nanoapertures, without damaging the surrounding metal nor the underlying SiO_2_ surface. The yield of subsequent QD-labelled DNA origami immobilisation on recycled substrates is not meaningfully affected by this cleaning process, remaining of ca 80% in single-dot patterning. Furthermore, if needed, only the DNA in the nanoapertures can be removed, leaving the QDs in the array. This can be achieved by exposing the substrate only to Ultraviolet/Ozone treatment without any subsequent sonication and/or rinsing step. Figure 4b shows that after such treatment most of the QDs remain in the nanoapertures, while the DNA nanostructures are successfully removed. In this case the overall final yield of single-QD patterning is slightly reduced to ~65%.

In summary, we have developed a reusable platform of general applicability for the assembly of individual nanoscale moieties with single-molecule control, in array configurations. As a proof of concept, we presented the patterning of individual CdSe/ZnS colloidal QDs on silicon and transparent insulating glass coverslips. Single-dot patterning was achieved via the use of DNA nanostructures as a scaffolding material, and their immobilisation in fabricated metal-based nanoaperture arrays. We demonstrated high level of control in the assembly of individual QDs (either one, two, or three) in nanoarray configurations, with a 60 nm interdot spacing within each cluster, and with a yield of up to 82% in single-dot patterning. The results presented here are specifically of interest for the development of single-QD based optoelectronic devices with applications in light harvesting, quantum information technology, data storage, and nanoscale optical circuitry. Additionally, this highly stable and reusable platform can be designed to exhibit different geometrical arrangements, and be employed for parallel single-molecule investigations of various nature, depending on the nanostructures employed.

## Methods

### DNA origami synthesis and modification

The triangular DNA origami is a single-layer trigonal DNA sheet with 120 nm side length. It is synthesized from 220 staples ssDNA strands (containing modified strands) and a 7249 bases ssDNA scaffold strand (M13mp18). Staple stands (Integrated DNA Technologies, 100 μM each in 1 × TAE buffer) and scaffold stand (single-stranded M13mp18, 1 μg/μL in Tris-HCL, Affymetrix) were mixed with a ratio of 5:1 with final concentration of 1 × TAE buffer, 12.5 mM Mg^2+^. The mixture was heated to 90 °C for 5 min and annealed from 90 °C to room temperature at the rate of 0.2 °C per min, which were completed by temperature controlled PCR machine (Hybaid Sprint PCR Thermal Cycler, Thermo Scientific). DNA origami then were purified and concentrated by using 100 kDa MWCO spin filters (Amicon^®^ Ultra, Ultracel-100 K, Millipore). The concentration was adjusted to 20 nM using a molecular weight of 330 g/mol per base and an extinction coefficient = 33 mg/ml for A260 = 1 in a NanoDrop Spectrophotometer (NanoVue™ Plus, GE Healthcare, UK). Different numbers of Quantum Dots were assembled on triangular DNA origami in order to reveal the pluripotent of this platform. The modification of different numbers of QDs were prepared by replacing normal staples strands on the outer edges of the DNA origami with biotinylated ssDNA staple strands which are called sticky ends. The staple strands on the inner edges of the triangular DNA origami were also replaced by amino modified ssDNA. After the assembly and purification procedure, QDs (Qdot^®^ 655 Streptavidin Conjugate, Life Technologies™) were assembled onto the DNA origami by biotin-streptavidin linkage by cooling down from 47 °C to room temperature in a PCR machine. We designed three different modified DNA origami: triangular DNA origami with one, two and three QDs (Figure SI2, Tables SI1&SI2).

### AFM imaging of DNA origami

DNA origami were checked under Atomic Force Microscopy (AFM, Bruker Dimension Icon) to confirm the synthesis and yield. 5 μL of triangular DNA origami in 1 × TAE-30 mM Mg^2+^ buffer was deposited onto freshly cleaved mica and left to adsorb to the surface for 2 min. Distilled water was used to wash the mica surface and samples were blown dry with compressed air. ScanAsyst™ mode (Dimension Icon with ScanAsyst, Bruker) in air was used with ScanAsyst-Air tips (silicon tip on Nitride lever, f_0_: 70 kHz, k: 0.4 N/m).

### FIB surface patterning

Freshly cleaned glass/silicon dioxide substrates (normal cleaning procedures: samples were soaked in Piranha solution for 5 min, then sonicated in ethanol for 10 min, sonicated in water for another 10 min, and cleaned with UV Ozone) were evaporated with ~1.5 nm chromium and ~3 nm gold layer on top. This is simpler than E-beam sample preparation, since there is no resist layer coating. We fabricated nanoapertures using Focus Ion Beam (FIB) on substrate surfaces. Each aperture of array is designed as 200 × 200 nm^2^ with ~1 μm spacing distance. Nanopatterned arrays were drawn in software and automatically run in the FEI^TM^ Quanta scanning electron microscope (SEM) and FIB system with a voltage/current of 30 kV/50 pA for the ion beam condition. The patterned surfaces were characterised with AFM and SEM with a voltage/current of 5.00 kV/107 pA and were cleaned with UV ozone prior to the covalent immobilisation of DNA origami.

### Covalent immobilisation

After purification, the DNA origami was diluted 20 times in Tris buffer (5 mM; pH 8.2) with 30 mM Mg^2+^. 60 μl of the DNA origami solution was cast on the substrate and placed in a 6-wells plate with moist Kimwipe. The sample was incubated for 90 minutes on a shaker. The sample was then washed with Tris buffer (5 mM; pH 8.2) with 30 mM Mg^2+^ (60 μl × 8). A 0.6 mM solution of carboxyethylsilane in the same Tris buffer was washed in with (60 μl × 8), and the sample was incubated for 2 minutes on a shaker. The buffer was then exchanged for MOPS buffer (10 mM; pH 8.1) with 30 mM Mg^2+^ (60 μl × 8). An equal volume of EDC (1-Ethyl-3-(3-dimethylaminopropyl) carbodiimide; 50 mM) and NHS (N-hydroxysulfosuccinimide; 100 mM) in the MOPS buffer was added to the sample’s volume and the sample was incubated for 10 minutes on a shaker. The sample was washed with the MOPS buffer, then rinsed with DPBS with 125 mM NaCl to remove any uncovalently bound structures, and subsequently rinsed with water. Finally, the sample was dipped in 25%, 50%, 75%, and 100% EtOH for 5 seconds each before being dried with compressed air. The samples were checked under AFM.

### Reuse of the substrate

The substrate can be reused via simple cleaning procedures. The substrates were treated by UV ozone and sonicated in a 60 °C water bath for 2 minutes. After rinsing with water, the substrates were ready for the covalent immobilisation. The yield of subsequent DNA immobilisation was found to be unaffected by the aforementioned cleaning procedure.

## Additional Information

**How to cite this article:** Huang, D. *et al*. DNA-Mediated Patterning of Single Quantum Dot Nanoarrays: A Reusable Platform for Single-Molecule Control. *Sci. Rep.* **7**, 45591; doi: 10.1038/srep45591 (2017).

**Publisher's note:** Springer Nature remains neutral with regard to jurisdictional claims in published maps and institutional affiliations.

## Supplementary Material

Supplementary Information
