# Supplementary material for: DNA-Mediated Patterning of Single Quantum Dot Nanoarrays: A Reusable Platform for Single-Molecule Control
Source: Sci Rep. 2017 Mar 28;7:45591. doi: 10.1038/srep45591 (PMC5368656; doi:10.1038/srep45591)
Supplement: Supplementary Information [file srep45591-s1.pdf]

DNA-Mediated Patterning of Single Quantum Dot Nanoarrays: A Reusable Platform for Single-Molecule Control

Da Huang [1], Mark Freeley [1] and Matteo Palma [1,*]

[1.] School of Biological and Chemical Sciences, Institute of Bioengineering, Queen Mary University of London, Mile End Road, London E1 4NS, United Kingdom

Supporting Information

I. DNA Origami

A. Self-Assembly of Triangular DNA Origami

The triangular DNA origami is a single-layer trigonal DNA sheet with 120nm side length. It is synthesized from 220 staples ssDNA strands (containing modified strands) and a 7249 bases ssDNA scaffold strand (M13mp18). Staple stands (Integrated DNA Technologies, 100μM each in 1× TAE buffer) and scaffold stand (single-stranded M13mp18, 1μg/μL in Tris-HCL, Affymetrix) were mixed with a ratio of 5:1 with final concentration of 1× TAE buffer, 12.5mM Mg^{2+}. The mixture was heated to 90℃ for 5 min and annealed from 90℃ to room temperature at the rate of 0.2℃ per min, which were completed by temperature controlled PCR machine (Hybaid Sprint PCR Thermal Cycler, Thermo Scientific). DNA origami then were purified and concentrated by using 100kDa MWCO spin filters (Amicon® Ultra, Ultracel-100K, Millipore). The concentration was adjusted to 20nM using a molecular weight of 330g/mol per base and an extinction coefficient =33mg/ml for A260=1 in a NanoDrop Spectrophotometer (NanoVue™ Plus, GE Healthcare, UK).

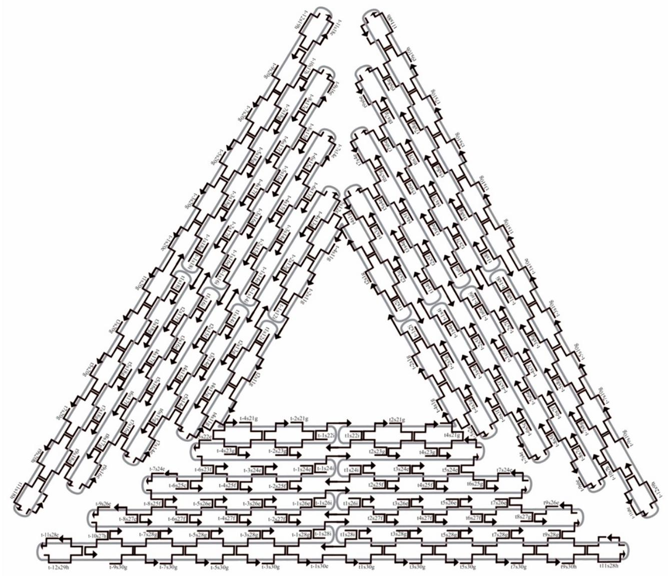

Figure SI1. Scheme of triangular DNA origami

B. Modification of DNA Origami

Different numbers of Quantum Dots were assembled on triangular DNA origami in order to reveal the pluripotent of this platform. The modification of different numbers of QDs were prepared by replacing normal staples strands on the outer edges of the DNA origami with biotinylated ssDNA staple strands which are called sticky ends. The staple strands on the inner edges of the triangular DNA origami were also replaced by amino modified ssDNA. After the assembly and purification procedure, QDs (Qdot® 655 Streptavidin Conjugate, Life Technologies™) were assembled onto the DNA origami by biotin-streptavidin linkage by cooling down from 47℃ to room temperature in a PCR machine. We designed three different modified DNA origami: triangular DNA origami with one, two and three QDs (Figure SI2, Table SI1&SI2).

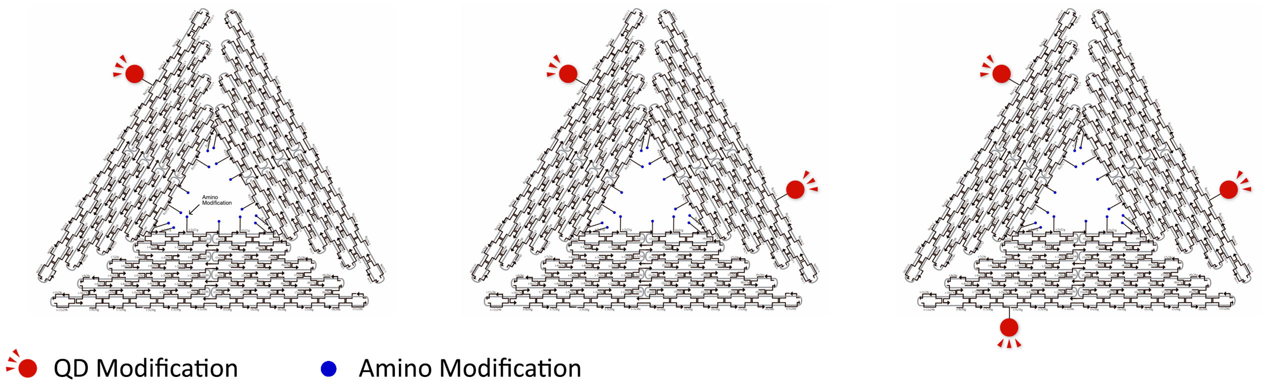

Figure SI2. Scheme of different number of QDs modified DNA origami: each DNA origami was designed to exhibit 15 amino modifications on the inner edges of the triangular nanostructure; these are indicated with blue circles in the figure. The red circles indicate the biotinylated ssDNA strands used as anchoring points for the attachment of streptavidinated QDs.

Table SI1. List of modified staple strands

**Oligo Name**	**Sequence**
Biotin-t5s8g	ACAAGAAAGCAAGCAAATCAGATAACAGCCATATTATTTATT-Biotin

Biotin-t5s18g	CCAAGCGCAGGCGCATAGGCTGGCAGAACTGGCTCATTATTT-Biotin
Biotin-t5s28g	TTAATGAAGTTTGATGGTGGTTCCGAGGTGCCGTAAAGCATT-Biotin
Amino-t1s2i	CCTTTTTTCATTTAACAATTTCATAGGATTAGTT-Amino
Amino-t1s12i	AGGGATAGCTCAGAGCCACCACCCCATGTCAATT-Amino
Amino-t1s22i	CGCGTCTGATAGGAACGCCATCAACTTTTACATT-Amino
Amino-t2s3g	TTTGATGATTAAGAGGCTGAGACTTGCTCAGTACCAGGCGTT-Amino
Amino-t2s13g	ACAGTCAAAGAGAATCGATGAACGACCCCGGTTGATAATCTT-Amino
Amino-ti2s13g	AGACGTTACCATGTACCGTAACACCCCTCAGAACCGCCACTT-Amino
Amino-t2s23g	AGAGTCAAAAATCAATATATGTGATGAAACAAACATCAAGTT-Amino
Amino-ti2s23g	TGGCAATTTTTAACGTCAGATGAAAACAATAACGGATTCGTT-Amino
Amino-tj2s23g	GTAACCGTCTTTCATCAACATTAAAATTTTTGTTAAATCATT-Amino
Amino-t4s3g	TTTAACGGTTCGGAACCTATTATTAGGGTTGATATAAGTATT-Amino
Amino-t4s13g	ACATAGCGCTGTAAATCGTCGCTATTCATTTCAATTACCTTT-Amino
Amino-ti4s13g	AGCGTAACTACAAACTACAACGCCTATCACCGTACTCAGGTT-Amino
Amino-t4s23g	CGTTCTAGTCAGGTCATTGCCTGACAGGAAGATTGTATAATT-Amino
Amino-ti4s23g	GATTATACACAGAAATAAAGAAATACCAAGTTACAAAATCTT-Amino
Amino-tj4s23g	GGATAGGTACCCGTCGGATTCTCCTAAACGTTAATATTTTTT-Amino

Table SI2. List of unmodified staple strands

**Oligo Name**	**Sequence**
t11s18h	AATACTGCGGAATCGTAGGGGGTAATAGTAAAATGTTTAGACT
t11s28h	TCTTTGATTAGTAATAGTCTGTCCATCACGCAAATTAACCGTT
t11s8h	CAGAAGGAAACCGAGGTTTTTAAGAAAAGTAAGCAGATAGCCG
t1s12i	TCATATGTGTAATCGTAAAACTAGTCATTTTC
t1s14i	GTGAGAAAATGTGTAGGTAAAGATACAACTTT
t1s16i	GGCATCAAATTTGGGGCGCGAGCTAGTTAAAG
t1s18i	TTCGAGCTAAGACTTCAAATATCGGGAACGAG
t1s22i	TCGGGAGATATACAGTAACAGTACAAATAATT
t1s24i	CCTGATTAAAGGAGCGGAATTATCTCGGCCTC

t1s26i	GCAAATCACCTCAATCAATATCTGCAGGTCGA
t1s28i	CGACCAGTACATTGGCAGATTCACCTGATTGC
t1s2i	CGGGGTTTCCTCAAGAGAAGGATTTTGAATTA
t1s4i	AGCGTCATGTCTCTGAATTTACCGACTACCTT
t1s6i	TTCATAATCCCCTTATTAGCGTTTTTCTTACC
t1s8i	ATGGTTTATGTCACAATCAATAGATATTAAAC
t2s11g	AGAAAAGCCCCAAAAAGAGTCTGGAGCAAACAATCACCAT
t2s13g	ACAGTCAAAGAGAATCGATGAACGACCCCGGTTGATAATC
t2s15f	ATAGTAGTATGCAATGCCTGAGTAGGCCGGAG
t2s17f	AACCAGACGTTTAGCTATATTTTCTTCTACTA
t2s1g	GATAAGTGCCGTCGAGCTGAAACATGAAAGTATACAGGAG
t2s21g	CCTGATTGCTTTGAATTGCGTAGATTTTCAGGCATCAATA
t2s25f	AAGGAATTACAAAGAAACCACCAGTCAGATGA
t2s27f	GGACATTCACCTCAAATATCAAACACAGTTGA
t2s5f	CCGGAACCCAGAATGGAAAGCGCAACATGGCT
t2s7f	AAAGACAACATTTTCGGTCATAGCCAAAATCA
t3s10g	GTCAGAGGGTAATTGATGGCAACATATAAAAGCGATTGAG
t3s14e	CAATATGACCCTCATATATTTTAAAGCATTAA
t3s16e	CATCCAATAAATGGTCAATAACCTCGGAAGCA
t3s18g	AACTCCAAGATTGCATCAAAAAGATAATGCAGATACATAA
t3s20g	CGCCAAAAGGAATTACAGTCAGAAGCAAAGCGCAGGTCAG
t3s24e	TAATCCTGATTATCATTTTGCGGAGAGGAAGG
t3s26e	TTATCTAAAGCATCACCTTGCTGATGGCCAAC
t3s28g	AGAGATAGTTTGACGCTCAATCGTACGTGCTTTCCTCGTT
t3s30g	AGAATCAGAGCGGGAGATGGAAATACCTACATAACCCTTC
t3s4e	TGTACTGGAAATCCTCATTAAAGCAGAGCCAC
t3s6e	CACCGGAAAGCGCGTTTTCATCGGAAGGGCGA
t3s8g	CATTCAACAAACGCAAAGACACCAGAACACCCTGAACAAA
t4s11g	GCAAATATTTAAATTGAGATCTACAAAGGCTACTGATAAA
t4s15f	CAGGCAAGATAAAAATTTTTAGAATATTCAAC

t4s17f	GATTAGAGATTAGATACATTTCGCAAATCATA
t4s1g	TAGCCCGGAATAGGTGAATGCCCCCTGCCTATGGTCAGTG
t4s21g	GCGCAGAGGCGAATTAATTATTTGCACGTAAATTCTGAAT
t4s25f	TAGGAGCATAAAAGTTTGAGTAACATTGTTTG
t4s27f	TGACCTGACAAATGAAAAATCTAAAATATCTT
t4s5f	CTCAGAGCATATTCACAAACAAATTAATAAGT
t4s7f	GGAGGGAATTTAGCGTCAGACTGTCCGCCTCC
t5s10g	GATAACCCACAAGAATGTTAGCAAACGTAGAAAATTATTC
t5s14e	TTAATGCCTTATTTCAACGCAAGGGCAAAGAA
t5s16e	TTAGCAAATAGATTTAGTTTGACCAGTACCTT
t5s18g	TAATTGCTTTACCCTGACTATTATGAGGCATAGTAAGAGC
t5s20g	AACACTATCATAACCCATCAAAAATCAGGTCTCCTTTTGA
t5s24e	AATGGAAGCGAACGTTATTAATTTCTAACAAC
t5s26e	TAATAGATCGCTGAGAGCCAGCAGAAGCGTAA
t5s28g	GAATACGTAACAGGAAAAACGCTCCTAAACAGGAGGCCGA
t5s30g	TTAAAGGGATTTTAGATACCGCCAGCCATTGCGGCACAGA
t5s4e	CCTTGAGTCAGACGATTGGCCTTGCGCCACCC
t5s6e	TCAGAACCCAGAATCAAGTTTGCCGGTAAATA
t5s8g	TTGACGGAAATACATACATAAAGGGCGCTAATATCAGAGA
t6s15g	ATAAAGCCTTTGCGGGAGAAGCCTGGAGAGGGTAG
t6s17f	TAAGAGGTCAATTCTGCGAACGAGATTAAGCA
t6s25g	TCAATAGATATTAAATCCTTTGCCGGTTAGAACCT
t6s27f	CAATATTTGCCTGCAACAGTGCCATAGAGCCG
t6s5g	CAGAGCCAGGAGGTTGAGGCAGGTAACAGTGCCCG
t6s7f	ATTAAAGGCCGTAATCAGTAGCGAGCCACCCT
t7s10g	ATAAGAGCAAGAAACATGGCATGATTAAGACTCCGACTTG
t7s14e	ATGACCCTGTAATACTTCAGAGCA
t7s16e	TAAAGCTATATAACAGTTGATTCCCATTTTTG
t7s18g	CGGATGGCACGAGAATGACCATAATCGTTTACCAGACGAC
t7s20g	GATAAAAACCAAAATATTAAACAGTTCAGAAATTAGAGCT

t7s24e	ACAATTCGACAACTCGTAATACAT
t7s26e	TTGAGGATGGTCAGTATTAACACCTTGAATGG
t7s28g	CTATTAGTATATCCAGAACAATATCAGGAACGGTACGCCA
t7s30g	GAATCCTGAGAAGTGTATCGGCCTTGCTGGTACTTTAATG
t7s4e	GCCGCCAGCATTGACACCACCCTC
t7s6e	AGAGCCGCACCATCGATAGCAGCATGAATTAT
t7s8g	CACCGTCACCTTATTACGCAGTATTGAGTTAAGCCCAATA
t8s17g	TAATTGCTTGGAAGTTTCATTCCAAATCGGTTGTA
t8s27g	CGCGAACTAAAACAGAGGTGAGGCTTAGAAGTATT
t8s7g	AGCCATTTAAACGTCACCAATGAACACCAGAACCA
t9s10h	TATCTTACCGAAGCCCAAACGCAATAATAACGAAAATCACCAG
t9s16e	ACTAAAGTACGGTGTCGAATATAA
t9s18g	TGCTGTAGATCCCCCTCAAATGCTGCGAGAGGCTTTTGCA
t9s20h	AAAGAAGTTTTGCCAGCATAAATATTCATTGACTCAACATGTT
t9s26e	ACCACCAGCAGAAGATGATAGCCC
t9s28g	TAAAACATTAGAAGAACTCAAACTTTTTATAATCAGTGAG
t9s30h	GCCACCGAGTAAAAGAACATCACTTGCCTGAGCGCCATTAAAA
t9s6e	CCATTAGCAAGGCCGGGGGAATTA
t9s8g	GAGCCAGCGAATACCCAAAAGAACATGAAATAGCAATAGC
t10s17h	ACCAACCTAAAAAATCAACGTAACAAATAAATTGGGCTTGAGA
t10s27h	AACTCACATTATTGAGTGTTGTTCCAGAAACCGTCTATCAGGG
t10s7h	ACGACAATAAATCCCGACTTGCGGGAGATCCTGAATCTTACCA
t12s19h	CCTGACGAGAAACACCAGAACGAGTAGGCTGCTCATTCAGTGA
t12s29h	ACGTGGACTCCAACGTCAAAGGGCGAATTTGGAACAAGAGTCC
t12s9h	TGCTATTTTGCACCCAGCTACAATTTTGTTTTGAAGCCTTAAA
t1s10e	AGAGAATAACATAAAAACAGGGAAGCGCATTA
t1s10g	AGAGAATAACATAAAAACAGGGAAGCGCATTA
t1s14e	ATTTTCTGTCAGCGGAGTGAGAATACCGATAT
t1s14i	CAACAGTTTATGGGATTTTGCTAATCAAAAGG
t1s16e	ATTCGGTCTGCGGGATCGTCACCCGAAATCCG

t1s16i	GCCGCTTTGCTGAGGCTTGCAGGGAAAAGGT
t1s18g	CGACCTGCGGTCAATCATAAGGGAACGGAACAACATTATT
t1s18i	GCGCAGACTCCATGTTACTTAGCCCGTTTTAA
t1s20e	ACAGGTAGAAAGATTCATCAGTTGAGATTTAG
t1s20g	AGAGAATAACATAAAAACAGGGAAGCGCATTA
t1s24e	CAGTTTGACGCACTCCAGCCAGCTAAACGACG
t1s24i	AGGAAGATGGGGACGACGACAGTAATCATATT
t1s26e	GCCAGTGCGATCCCCGGGTACCGAGTTTTTCT
t1s26i	CTCTAGAGCAAGCTTGCATGCCTGGTCAGTTG
t1s28g	TTTCACCAGCCTGGCCCTGAGAGAAAGCCGGCGAACGTGG
t1s28i	CCTTCACCGTGAGACGGGCAACAGCAGTCACA
t1s30g	AGAGAATAACATAAAAACAGGGAAGCGCATTA
t1s30e	CGAGAAAGGAAGGGAAGCGTACTATGGTTGCT
t1s4e	TTATCAAACCGGCTTAGGTTGGGTAAGCCTGT
t1s4i	TTTAACCTATCATAGGTCTGAGAGTTCCAGTA
t1s6e	TTAGTATCGCCAACGCTCAACAGTCGGCTGTC
t1s6i	AGTATAAAATATGCGTTATACAAAGCCATCTT
t1s8g	TTTCCTTAGCACTCATCGAGAACAATAGCAGCCTTTACAG
t1s8i	CAAGTACCTCATTCCAAGAACGGGAAATTCAT
t2s11g	CCTCAGAACCGCCACCCAAGCCCAATAGGAACGTAAATGA
t2s15f	CACGCATAAGAAAGGAACAACTAAGTCTTTCC
t2s17f	ATTGTGTCTCAGCAGCGAAAGACACCATCGCC
t2s1g	AAAACAAAATTAATTAAATGGAAACAGTACATTAGTGAAT
t2s21g	GCTCATTTTTTAACCAGCCTTCCTGTAGCCAGGCATCTGC
t2s23g	GTAACCGTCTTTCATCAACATTAAAATTTTTGTTAAATCA
t2s25f	ACGTTGTATTCCGGCACCGCTTCTGGCGCATC
t2s27f	CCAGGGTGGCTCGAATTCGTAATCCAGTCACG
t2s5f	ACTAGAAATATATAACTATATGTACGCTGAGA
t2s7f	TCAATAATAGGGCTTAATTGAGAATCATAATT
t3s10g	AACGTCAAAAATGAAAGCAAGCCGTTTTTATGAAACCAA

t3s14e	GTTTTGTCAGGAATTGCGAATAATCCGACAAT
t3s16e	GACAACAAGCATCGGAACGAGGGTGAGATTTG
t3s18g	TATCATCGTTGAAAGAGGACAGATGGAAGAAAAATCTACG
t3s20g	TTAATAAAACGAACTAACCGAACTGACCAACTCCTGATAA
t3s24e	TGTAGATGGGTGCCGGAAACCAGGAACGCCAG
t3s26e	GGTTTTCCATGGTCATAGCTGTTTGAGAGGCG
t3s28g	GTTTGCGTCACGCTGGTTTGCCCCAAGGGAGCCCCCGATT
t3s30g	TAGAGCTTGACGGGGAGTTGCAGCAAGCGGTCATTGGGCG
t3s4e	GATTAAGAAATGCTGATGCAAATCAGAATAAA
t3s6e	CACCGGAATCGCCATATTTAACAAAATTTACG
t3s8g	AGCATGTATTTCATCGTAGGAATCAAACGATTTTTTGTTT
t4s11g	AGGTTTAGTACCGCCATGAGTTTCGTCACCAGGATCTAAA
t4s15f	TAGTTGCGAATTTTTTCACGTTGATCATAGTT
t4s17f	GTACAACGAGCAACGGCTACAGAGGATACCGA
t4s1g	GAGCAAAAGAAGATGAGTGAATAACCTTGCTTATAGCTTA
t4s21g	GTTAAAATTCGCATTAATGTGAGCGAGTAACACACGTTGG
t4s25f	AGTTGGGTCAAAGCGCCATTCGCCCCGTAATG
t4s27f	CGCGCGGGCCTGTGTGAAATTGTTGGCGATTA
t4s3g	ACATAGCGCTGTAAATCGTCGCTATTCATTTCAATTACCT
t4s5f	GTTAAATACAATCGCAAGACAAAGCCTTGAAA
t4s7f	CCCATCCTCGCCAACATGTAATTTAATAAGGC
t5s10g	TCCCAATCCAAATAAGATTACCGCGCCCAATAAATAATAT
t5s16e	AACAGCTTGCTTTGAGGACTAAAGCGATTATA
t5s20g	ACCAGTCAGGACGTTGGAACGGTGTACAGACCGAAACAAA
t5s26e	TGCTGCAAATCCGCTCACAATTCCCAGCTGCA
t5s30g	CTAAATCGGAACCCTAAGCAGGCGAAAATCCTTCGGCCAA
t5s6e	GTGTGATAAGGCAGAGGCATTTTCAGTCCTGA
t6s13f	ACAGACAGCCCAAATCTCCAAAAAAAAATTTCTTA
t6s15c	CGAGGTGAGGCTCCAAAAGGAGCC
t6s17f	ACCCCCAGACTTTTTCATGAGGAACTTGCTTT

t6s23f	CGGCGGATTGAATTCAGGCTGCGCAACGGGGGATG
t6s25c	TGGCGAAATGTTGGGAAGGGCGAT
t6s27f	TGTCGTGCACACAACATACGAGCCACGCCAGC
t6s3f	TCCCTTAGAATAACGCGAGAAAACTTTTACCGACC
t6s5c	GTTTGAAATTCAAATATATTTTAG
t6s7f	AATAGATAGAGCCAGTAATAAGAGATTTAATG
t7s10g	GCCAGTTACAAAATAATAGAAGGCTTATCCGGTTATCAAC
t7s18g	AAAACACTTAATCTTGACAAGAACTTAATCATTGTGAATT
t7s20g	ACCTTATGCGATTTTATGACCTTCATCAAGAGCATCTTTG
t7s28g	TTCCAGTCCTTATAAATCAAAAGAGAACCATCACCCAAAT
t7s30g	CAAGTTTTTTGGGGTCGAAATCGGCAAAATCCGGGAAACC
t7s8g	GCGCCTGTTATTCTAAGAACGCGATTCCAGAGCCTAATTT
t8s15f	CGGTTTATCAGGTTTCCATTAAACGGGAATACACT
t8s17c	GGCAAAAGTAAAATACGTAATGCC
t8s25f	TCTTCGCTATTGGAAGCATAAAGTGTATGCCCGCT
t8s27c	GCGCTCACAAGCCTGGGGTGCCTA
t8s5f	TTCTGACCTAAAATATAAAGTACCGACTGCAGAAC
t8s7c	TCAGCTAAAAAAGGTAAAGTAATT
t9s10g	ACGCTAACGAGCGTCTGGCGTTTTAGCGAACCCAACATGT
t9s20g	TGGTTTAATTTCAACTCGGATATTCATTACCCACGAAAGA
t9s30g	CGATGGCCCACTACGTATAGCCCGAGATAGGGATTGCGTT
tsrem1	GCGCTTAATGCGCCGCTACAGGGC
t5s2et6s23c3T	TTAATTAATTTTTTACCATATCAAA
t7s4et8s25c2T	TTAATTTCATCTTAGACTTTACAA
t9s6et10s27c1T	CTGTCCAGACGTATACCGAACGA
t11s8et12s29c0T	TCAAGATTAGTGTAGCAATACT
t5s12et6s3c3T	TGTAGCATTCCTTTTATAAACAGTT
t7s14et8s5c2T	TTTAATTGTATTTCCACCAGAGCC
t9s16et10s7c1T	ACTACGAAGGCTTAGCACCATTA
t11s18et12s9c0T	ATAAGGCTTGCAACAAAGTTAC

t5s22et6s13c-T	GTGGGAACAAATTTCTATTTTTGAG
t7s24et8s15c2T	CGGTGCGGGCCTTCCAAAAACATT
t9s26et10s17c1T	ATGAGTGAGCTTTTAAATATGCA
t11s28et12s19c0T	ACTATTAAAGAGGATAGCGTCC

C. AFM Imaging of DNA origami

DNA origami were checked under Atomic Force Microscopy (AFM, Bruker Dimension Icon) to confirm the synthesis and yield. 5μL of triangular DNA origami in 1× TAE-30 mM Mg^{2+} buffer was deposited onto freshly cleaved mica and left to adsorb to the surface for 2 min. Distilled water was used to wash the mica surface and samples were blown dry with compressed air. ScanAsyst™ mode (Dimension Icon with ScanAsyst, Bruker) in air was used with ScanAsyst-Air tips (silicon tip on Nitride lever, f_0: 70kHz, k: 0.4N/m).

D. Analysis of DNA origami

The yields of modified DNA origami were counted via AFM images. AFM images with a 4×4μm² dimension were analysed for each kind of modified DNA origami (Figure SI3). We achieved 90.9% of yield for DNA origami with one QD, 89.4% of yield for DNA origami with two QDs and 85.7% of yield for DNA origami with three QDs.

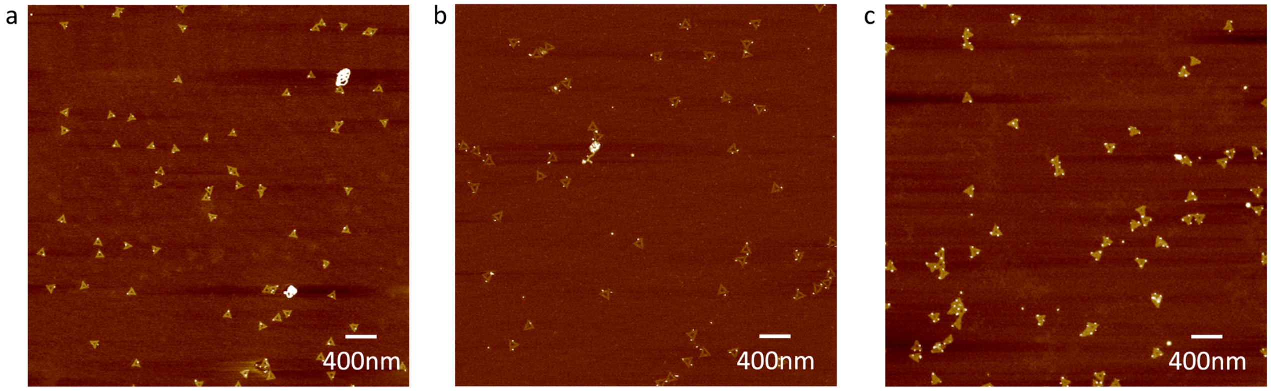

Figure SI3. AFM images of modified DNA origami, including DNA origami with one QD (a), two QDs (b) and three QDs (c).

II. Surface Patterning

A. FIB Surface Patterning

Freshly cleaned glass/silicon dioxide substrates (normal cleaning procedures: samples were soaked in Piranha solution for 5min, then sonicated in ethanol for 10min, sonicated in water for another 10min, and cleaned with UV Ozone) were evaporated with ~1.5nm chromium and ~3nm gold layer on top. This is simpler than E-beam sample preparation, since there is no resist layer coating. We fabricated nanoapertures using Focus Ion Beam (FIB) on substrate surfaces. Each aperture of array is designed as $200\times200nm^2$ with ~1 μm spacing distance. Nanopatterned arrays were drawn in software and automatically run in the FEI™ Quanta scanning electron microscope (SEM) and FIB system with a voltage/current of 30kV/50pA for the ion beam condition. The patterned surfaces were characterised with AFM and SEM with a voltage/current of 5.00kV/107pA and were cleaned with UV ozone prior to the covalent immobilisation of DNA origami.

B. Covalent Immobilisation

After purification, the DNA origami was diluted 20 times in Tris buffer (5mM; pH 8.2) with 30mM Mg^{2+}. 60μl of the DNA origami solution was cast on the substrate and placed in a 6-wells plate with moist Kimwipe. The sample was incubated for 90 minutes on a shaker. The sample was then washed with Tris buffer (5mM; pH 8.2) with 30mM Mg^{2+} (60μl x 8). A 0.01% solution of carboxyethylsilane in the same Tris buffer was washed in with (60μl x 8), and the sample was incubated for 2 minutes on a shaker. The buffer was then exchanged for MOPS buffer (10mM; pH 8.1) with 30mM Mg^{2+} (60μl x 8). An equal volume of EDC (1-Ethyl-3-(3-dimethylaminopropyl) carbodiimide; 50mM) and NHS (N-hydroxysulfosuccinimide; 100mM) in the MOPS buffer was added to the sample's volume and the sample was incubated for 10 minutes on a shaker. The sample was washed with the MOPS buffer, then rinsed with DPBS with 125mM NaCl to remove any uncovalently bound structures, and subsequently rinsed with water. Finally, the sample was dipped in 25%, 50%, 75%, and 100% EtOH for 5 seconds each before being dried with compressed air. The samples were checked under AFM.

C. Analysis of Covalent Immobilisation

The yields of covalent immobilisation were counted via AFM images. AFM images of apertures were analysed for each kind of modified DNA origami (Figure SI4). We achieved 90.6% of yield for DNA origami with one QD, 89.06% of yield for DNA origami with two QDs and 92.2% of yield for DNA origami with three QDs.

D. Reuse of the Substrate

The substrate can be reused via simple cleaning procedures. The substrates were treated by UV ozone and sonicated in a 60℃ water bath for 2 minutes. After rinsing with water the substrates were ready for the covalent immobilisation. The yield of subsequent DNA immobilisation was found to be unaffected by the aforementioned cleaning procedure.

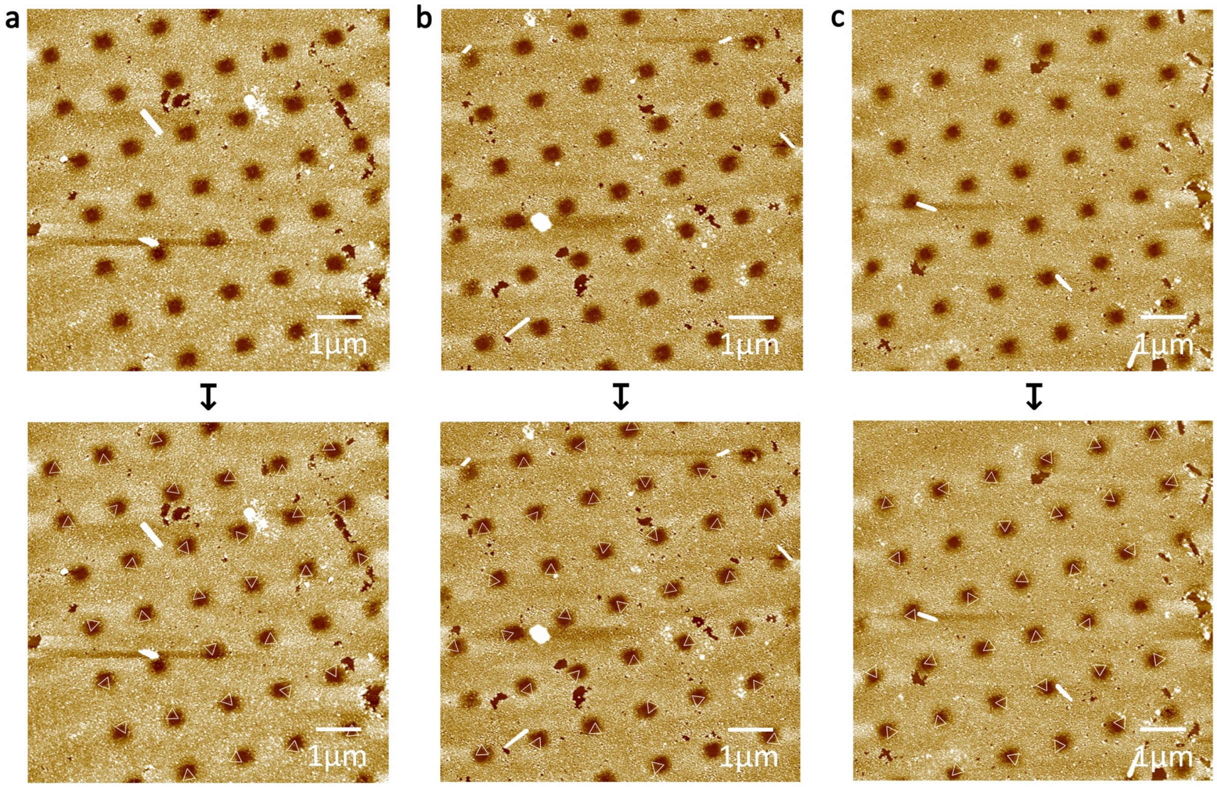

Figure SI4. AFM images of covalent immobilisation. The triangular outlines show the location of each DNA origami. Different numbers of QDs modified DNA origami are shown: (a) DNA origami with three QDs, (b) DNA origami with two QDs, and (c) DNA origami with one QD.
